# Real‐world outcomes of various regimens of recombinant human endostatin combined with chemotherapy in non‐driver gene mutation advanced non‐small cell lung cancer

**DOI:** 10.1002/cam4.2014

**Published:** 2019-02-14

**Authors:** Zhongtai Wang, Hui Zhang, Chunhua Zhou, Xiaoyan Long, Rui Guan, Nong Yang, Yongchang Zhang

**Affiliations:** ^1^ Department of Medical Oncology, Lung Cancer and Gastrointestinal Unit Hunan Cancer Hospital/The Affiliated Cancer Hospital of Xiangya School of Medicine Central South University Changsha China; ^2^ Graduate Schools University of South China Hengyang Hunan China

**Keywords:** chemotherapy, different administration, non‐small cell lung cancer, real‐world study, recombinant human‐endostatin

## Abstract

**Aims:**

This real‐world study is conducted to evaluate the efficacy and safety of recombinant human endostatin (rh‐endostatin) combined with chemotherapy as first‐line treatment for non‐driver genes mutation non‐small cell lung cancer (NSCLC) patients, and establish evidence‐based optimal regimen for rh‐endostatin.

**Patients and Methods:**

Using propensity score matching (cut‐off: 0.01), 88 patients were eligible for our study, 34 of which received platinum‐based chemotherapy alone (chemotherapy group), 54 patients received platinum‐based chemotherapy plus rh‐endostatin (rh‐endostatin group). Among those 54 patients in the rh‐endostatin group, 27 patients received rh‐endostatin administered at 7.5 mg/m^2^ from day 1 to day 14 (rh‐endostatin 14d group), and the other 27 patients were administered at 15 mg/m^2^ from day 1 to day 7 (rh‐endostatin 7d group). The primary endpoint was progression‐free survival (PFS) and secondary endpoints were overall survival (OS), overall response rate (ORR), disease control rate (DCR), and safety.

**Results:**

There were no differences in clinic characteristics among 3 groups. Compared with chemotherapy group, rh‐endostatin group improved PFS and OS significantly. The median PFS was 6 months vs 4.5 months (*P* = 0.047), and median OS was 20 months vs 10 months (*P* < 0.001). The ORR was 33.3% vs 20.6% (*P* = 0.197) and DCR was 83.3% vs 64.7% (*P* = 0.046) in the rh‐endostatin group and chemotherapy group, respectively. The comparisons between the rh‐endostatin 7d and 14d groups revealed a significant improvement in PFS for the rh‐endostatin 7d group (*P* = 0.044), but no significant differences in OS (*P* = 0.111), ORR (*P* = 0.074), or DCR (*P* = 0.234). The incidences of grade 3 and 4 adverse events were similar among 3 groups.

**Conclusion:**

Chemotherapy combined with rh‐endostatin was more effective than chemotherapy alone for non‐driver gene mutation NSCLC patients. The administration of rh‐endostatin for 7 days at 15 mg/m^2^ was non‐inferior to 14 days at 7.5 mg/m^2^ in prolonging patients’ PFS. Further evaluation should be conducted before its application in clinical work.

## 1 INTRODUCTION

Non‐small cell lung cancer (NSCLC) is the most common type of epithelial lung accounting for nearly 85% of all lung cancer patients. Its cancer incidence and mortality are the highest worldwide and the 5‐year overall survival (OS) of patients with advanced NSCLC is <5%.^1^ Currently, the development of platinum‐based chemotherapy mostly are limited to better drug tolerance and less toxic side effects, but the progress in efficacy is inadequate. In past decades, great clinical improvements have been achieved in NSCLC with the participation of target therapy like EGFR tyrosine kinase inhibitors.^2^, ^3^ However, patients with non‐driver genes have shown far less clinical response to those target therapy.

Recombinant human endostatin (rh‐endostatin) inhibits the proliferation of vascular endothelial cells through multiple targets, thereby suppressing angiogenesis and tumor growth.^4^ It has been reported that the combination of rh‐endostatin with cisplatin/vinorelbine significantly increased time to progression and overall response rate (ORR) in NSCLC patients.^5^ On the basis of this study, the China Food and Drug Administration (CFDA) approved rh‐endostatin as the first‐line treatment for advanced NSCLC patients in 2005. After that the efficacy of rh‐endostatin has been proved in several researches.^6^, ^7^, ^8^ Rh‐endostatin in those studies was administered intravenously at 7.5 mg/m^2^ daily from day 1 to 14 every 3 weeks, which has been widely used in clinical practice. However, the administration for 14 days could result in several problems, including increasing hospital stay and cost, leading to a lower compliance of patients and reduced treatment response. Some researchers later adjusted the dose of rh‐endostatin to 15 mg/m^2^ from day 1 to 7 every 3 weeks to solve the problem.

We conducted this project to investigate the routine practice different administration rh‐endostatin combined with chemotherapy as first‐line treatment in advanced non‐driver gene mutation NSCLC patients. Also we investigated effect of different administration modes on patient outcome.

## 2 PATIENTS AND METHODS

### 2.1 Patients

Between April 2014 and April 2017, 136 advanced NSCLC patients who received first‐line chemotherapy combined with rh‐endostatin at Hunan Cancer Hospital were enrolled in this study. All patients were ≥18 years old and histologically diagnosed with inoperable stage III or IV NSCLC, with an Eastern Cooperative Oncology Group (ECOG) performance status (PS) of 0‐3. Patients with hepatic or renal dysfunction and cardiac disease were excluded. We used propensity score matching (PSM) to normalize the baseline characteristics among the 3 groups. The characteristics of the patients including sex, age, ECOG PS, smoking history, histological grade, pathology, and metastasis were listed in Table 1. All the patients signed written informed consent. The study was approved by Hunan Cancer Hospital Ethic Committee. The study was also conducted in accordance with the Declaration of Helsinki.

**Table 1:** Patients’ characteristics

Variables	Chemotherapy alone group n (%)	Rh‐endostatin 14d group n (%)	Rh‐endostatin 7d group n (%)	*P*
Total	34	27	27	
Age, years				
Median	57	59	59	
Range	41‐68	39‐78	44‐69	
≥65	9 (26.5)	4 (14.8)	8 (29.6)	0.399
＜65	25 (73.5)	23 (85.2)	19 (70.4)	
Sex				
Male	27 (79.4)	18 (66.7)	24 (88.9)	0.137
Female	7 (20.6)	9 (33.3)	3 (11.1)	
ECOG PS				
0, 1	33 (97.0)	27 (100.0)	27 (100.0)	0.448
2, 3	1 (3.0)	0 (0)	0 (0)	
Smoke				
Yes	24 (70.6)	17 (63.0)	22 (81.5)	0.316
No	10 (29.4)	10 (37.0)	5 (18.5)	
Grade				
III	15 (44.1)	10 (37.0)	14 (51.9)	0.548
Ⅳ	19 (55.9)	17 (63.0)	13 (48.1)	
Pathology				
AD	8 (23.5)	9 (33.3)	3 (11.1)	0.215
SCC	24 (70.6)	17 (63.0)	24 (88.9)	
Other	2 (5.9)	1 (3.7)	0 (0)	
Metastasis				
Lung	3 (8.8)	4 (14.8)	4 (14.8)	0.264
Liver	7 (20.6)	2 (7.4)	1 (3.7)	
Bone	8 (23.5)	6 (22.2)	6 (22.2)	
Brain	4 (11.8)	5 (18.5)	1 (3.7)	
Other	5 (14.7)	11 (40.7)	8 (29.6)	
None	16 (47.1)	9 (26.5)	14 (51.9)	

AD, adenocarcinoma; ECOG, eastern cooperative oncology group; PS, performance status; SCC, squamous cell carcinoma.

### 2.2 Treatment

After selection through PSM, 34 patients only received platinum‐based chemotherapy every 21 days was set as chemotherapy alone group. Fifty‐four patients received platinum‐based chemotherapy plus rh‐endostatin was set as rh‐endostatin group. Among those 54 patients, 27 patients (rh‐endostatin 14d group) received platinum‐based chemotherapy plus rh‐endostatin administered daily at 7.5 mg/d, from day 1 to 14 every 21 days (d1‐14, q21d), and 27 patients (rh‐endostatin 7d group) received chemotherapy plus rh‐endostatin administered daily at 15 mg/d, from day 1 to 7 every 21 days (d1‐7, q21d). All chemotherapy regimens in this study included TP: paclitaxel (175 mg/m^2^, d1) + cisplatin (100 mg/m^2^, d1), TC: paclitaxel + carboplatin (area under the curve, 6), GP: gemcitabine (1250 mg/m^2^, d1, d8) + cisplatin, GC: gemcitabine + carboplatin, PP: pemetrexed (500 mg/m^2^, d1) + cisplatin, and PC: pemetrexed + carboplatin. Treatment will continue until progressive disease (PD), consent withdrawal or intolerable toxicity.

### 2.3 Assessment

Patients were evaluated for response by computed tomography every 2 treatment cycles. Treatment response was evaluated as complete response (CR), partial response (PR), stable disease (SD), PD, or not evaluable according to the Response Evaluation Criteria in Solid Tumor criteria 1.1.^9^ The ORR was defined as the sum of CR and PR. The disease control rate (DCR) was defined as the sum of CR, PR, and SD. Toxicities were graded according to the National Cancer Institute Common Terminology Criteria for Adverse Events version 5.0. Informed consent was obtained from all patients. The primary endpoint was progression‐free survival (PFS). Secondary endpoints were OS, ORR, and DCR.

### 2.4 Statistics analysis

The proportions in 3 groups and treatment responses were compared by the χ^2^ test. Survival distributions were estimated with the Kaplan‐Meier method and compared using the log‐rank test. All statistical analyses were performed using the SPSS 22.0 software for Windows (SPSS Corp., Armonk, NY, USA); *P* < 0.05 was considered to indicate a statistically significant difference.

## 3 RESULTS

### 3.1 Patient characteristics

The characteristics of the patients, including age, sex, performance, smoking status, grade, pathology, and metastasis, which are similar among the 3 groups, are summarized in Table 1. A total of 21 patients (23.9%) were 65 years old or older, 19 patients (21.6%) were female, and 25 patients (28.4%) had never smoked. Only 1 patient (1.1%) had a poor PS (2). According to the TNM classification for NSCLC patients (seventh edition), 39 patients (44.3%) were at stage III, and 49 patients (55.7%) were at stage IV. Twenty (22.7%) patients were diagnosed with adenocarcinoma, and 65 (73.9%) patients with squamous cell carcinoma.

### 3.2 Treatment response

The treatment responses are listed in Table 2. The ORR and DCR in the chemotherapy group were 20.6% and 64.7% vs 33.3% and 83.3% in the rh‐endostatin group, respectively. The difference of ORR was no statistical significance (*P = *0.197), but the DCR was significantly improved in the rh‐endostatin group (*P* = 0.046). The ORR and DCR in the rh‐endostatin 14d group were 44.4% and 88.9%, vs 22.2% and 77.8% in the rh‐endostatin 7d group, respectively. No significant differences were observed in the comparison between the 2 groups (ORR: *P* = 0.074, DCR: *P* = 0.234). No patient achieved CR in the whole population.

**Table 2:** Summary of treatment efficacy

Response	Chemotherapy group n (%)	Rh‐endostatin group n (%)	*P*	Rh‐endostatin group n (%)		
				Rh‐endostatin 14d group n (%)	Rh‐endostatin 7d group n (%)	*P*
CR	0 (0)	0 (0)	—	0 (0)	0 (0)	—
PR	7 (20.6)	18 (33.3)	0.1970	12 (44.4)	6 (22.2)	0.07
ORR	7 (20.6)	18 (33.3)	0.1970	12 (44.4)	6 (22.2)	0.07
SD	15 (44.1)	27 (50.0)	0.5910	12 (44.4)	15 (55.6)	0.29
DCR	22 (64.7)	45 (83.3)	0.0460	24 (88.9)	21 (77.8)	0.23
PD	6 (17.6)	2 (3.7)	0.0250	1 (3.7)	1 (3.7)	0.76
NE	6 (17.6)	7 (13.0)	—	2 (7.4)	5 (18.5)	—

CR, complete response; DCR, disease control rate; NE, not evaluable; ORR, overall response rate; PD, progressive disease; PR, partial response; SD, stable disease.

### 3.3 Survival

**Figure 1:**
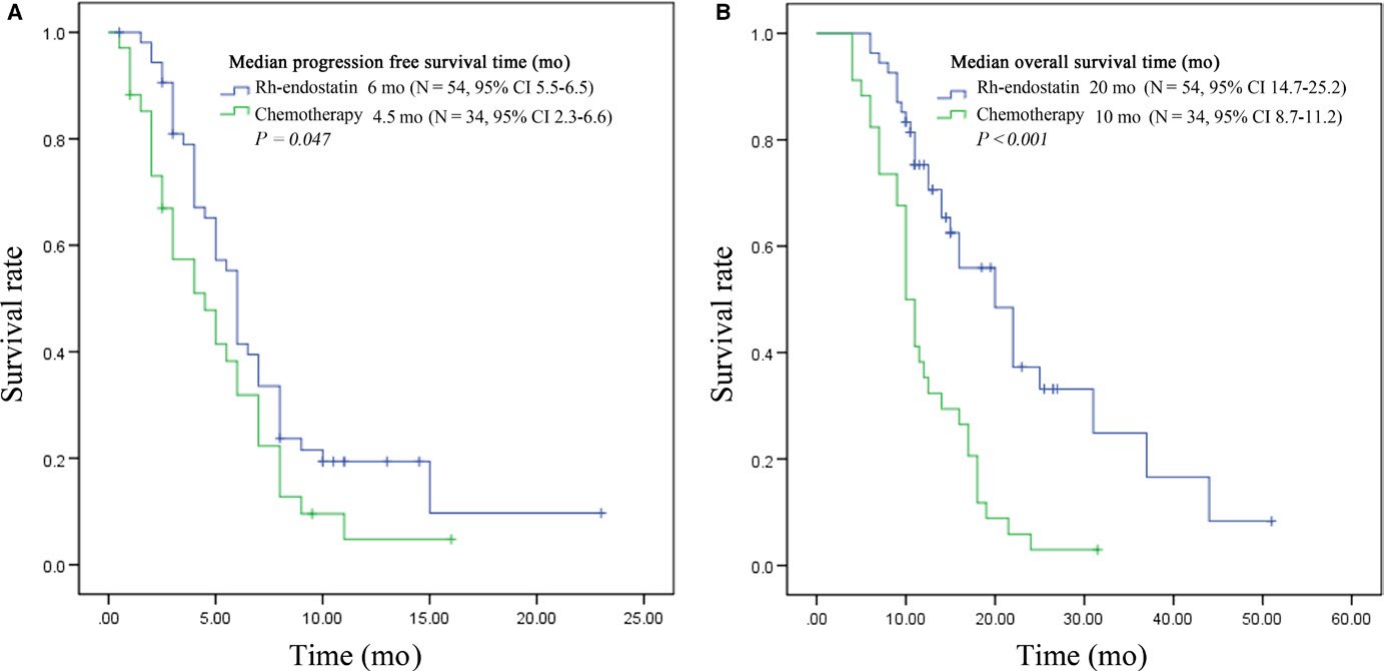
Plot of Kaplan‐Meier estimates for progression‐free survival time (A) and overall survival time (B) for the recombinant human endostatin (Rh‐endostatin) group compared with chemotherapy group

**Figure 2:**
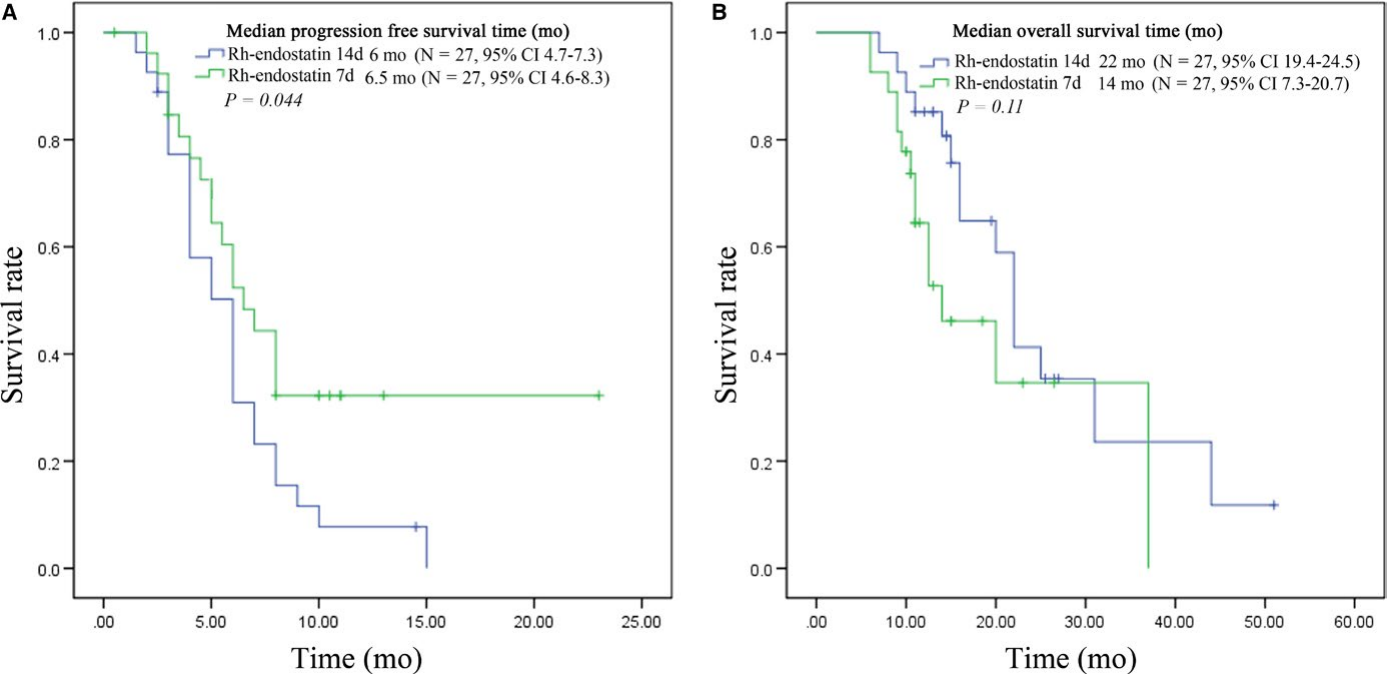
Plot of Kaplan‐Meier estimates for progression‐free survival time (A) and overall survival time (B) for the endostar 7d group compared with recombinant human endostatin (Rh‐endostatin) 14d group

The median PFS in the rh‐endostatin group was 6.0 months vs 4.5 months in the chemotherapy group (*P* = 0.047, Figure 1A) and the median OS in the rh‐endostatin group was 20.0 months vs 10.0 months in the chemotherapy group (*P *< 0.001; Figure 1B). Both PFS and OS were longer in rh‐endostatin group and of statistical significance. Also the 1 year survival rate in the chemotherapy alone and rh‐endostatin group was 71.47%, higher than chemotherapy group of 41.18% (and 71.47% respectively, *P *< 0.01). In addition, there was a significant advantage in the PFS of the rh‐endostatin 7d group compared with that of the rh‐endostatin 14d group (6.5 months vs 6.0 months; *P* = 0.044, Figure 2A). Although a noticeable improvement was detected in the OS of the rh‐endostatin 14d group compared to 7d group, there was no significant difference (22 months vs 14 months; *P* = 0.111; Figure 2B).

### 3.4 Toxicity

Among the 88 patients enrolled, 16 (18.2%) had severe adverse events (grade 3‐4). In addition, the incidence of severe myelosuppression in the chemotherapy group, the rh‐endostatin 14d group, and the rh‐endostatin 7d groups was 11.6%, 22.2%, and 3.7%, respectively. Common severe adverse reactions included 3 cases of leukopenia, 3 cases of neutropenia, 4 cases of thrombocytopenia, and 3 cases of vomiting. The incidence of each severe adverse reaction was lower than 10% in all groups. Other rare adverse reactions included mild liver damage in 2 cases, mild constipation in 2 cases, and fatigue in 1 case. Details of these adverse reactions are given in Table 3. In the chemotherapy group, there were a total of 16 cases (47.1%) of adverse events and 7 cases (20.6%) of serious adverse events. Anemia was the most common adverse reaction, including 3 cases (8.8%) of mild to moderate anemia and 1 case (2.9%) of severe anemia. In the rh‐endostatin 14d group, there were a total of 41 cases of adverse events, most of which were mild‐to‐moderate (Grades 1‐2) adverse events, and 15 patients had mild nausea with an incidence rate of 55.6%. These were the most common adverse reactions. Mild vomiting occurred in 6 patients with an incidence rate of 22.2%. Serious adverse events occurred in 6 patients (22.2%), including 2 patients with leukopenia (7.4%), 2 patients with granulocytopenia (7.4%), and 2 patients with thrombocytopenia (7.4%). In the rh‐endostatin 7d group, a total of 16 cases (59.3%) of adverse reactions occurred, and 3 cases (11.1%) of serious adverse events, including 1 case of grade 4 thrombocytopenia (3.7%), 1 case of nausea (3.7%), and 1 case of vomiting (3.7%). Compared with the rh‐endostatin 14d group, the incidence of gastrointestinal adverse reactions and severe myelosuppression was lower in the rh‐endostatin 7d group.

**Table 3:** Treatment related adverse events

Adverse events	Chemotherapy group n (%)		Rh‐endostatin 14d group n (%)		Rh‐endostatin 7d group n (%)	
	Grade 1‐2	Grade 3‐4	Grade 1‐2	Grade 3‐4	Grade 1‐2	Grade 3‐4
Leukopenia	5 (14.7)	6 (17.6)	8 (29.6)	7 (25.9)	6 (22.2)	7 (25.9)
Neutropenia	5 (14.7)	5 (14.7)	7 (25.9)	5 (18.5)	5 (18.5)	4 (14.8)
Anemia	3 (8.8)	1 (2.9)	1 (3.7)	1 (3.7)	1 (3.7)	2 (7.4)
Thrombocytopenia	2 (5.9)	1 (2.9)	3 (11.1)	2 (7.4)	0 (0)	1 (3.7)
Nausea	1 (2.9)	1 (2.9)	15 (55.6)	2 (7.4)	3 (11.1)	1 (3.7)
Fatigue	0 (0)	0 (0)	0 (0)	0 (0)	1 (3.7)	0 (0)
Vomiting	1 (2.9)	2 (5.9)	6 (22.2)	0 (0)	0 (0)	1 (3.7)
Liver dysfunction	1 (2.9)	0 (0)	2 (7.4)	0 (0)	0 (0)	0 (0)
Constipation	0 (0)	0 (0)	2 (7.4)	0 (0)	0 (0)	0 (0)
Hemoptysis	0 (0)	0 (0)	2 (7.4)	0 (0)	2 (7.4)	1(3.7)
Cardiotoxicity	0 (0)	0 (0)	3 (11.1)	0 (0)	2 (7.4)	0 (0)
Hypertension	0 (0)	0 (0)	0 (0)	0 (0)	0 (0)	0 (0)
Proteinuria	0 (0)	0 (0)	0 (0)	0 (0)	0 (0)	0 (0)

## 4 DISCUSSION

Endostatin was a 20 kDa c‐terminal fragment of collagen XVIII,^10^ originally isolated by O’ Reilly from supernatant of a murine hemangio‐endothelioma.^11^ In preclinical studies, endostatin was demonstrated to play key role in anti‐angiogenesis by normalizing tumor microvessels and inhibiting the activities of vascular endothelial cells via multiple targets.^4^, ^12^ It showed a strong suppression on various murine tumors growth in vivo, such as ovarian cancer,^13^ renal cell cancer,^14^ JC breast carcinoma,^15^ Lewis lung carcinoma, T241 fibrosarcoma, and B16F10 melanoma.^10^

Rh‐endostatin (rh‐endostatin) is a new and modified recombined human endostatin developed by Chinese scientist, which had been demonstrated to offer clinical benefits for patients with advanced NSCLC in several clinical trials.^6^, ^16^, ^17^, ^18^ Based on the encouraging outcomes in a large randomized, double‐blind phase III clinical trial,^19^ rh‐endostatin was approved by CFDA in combination with vinorelbine/cisplatin for patients with advanced NSCLC in 2005. In recent years, Chinese scholars have begun to investigate rh‐endostatin in greater extent for the further improvement in efficacy. He et al^20^ have reported that the expression of regulator of G–protein signaling 5 (RGS5) and carbonic anhydrase 9 (CA) were reduced during the vascular normalization window, which suggested that RGS5 and CA may be the key to define the optimal administration time of rh‐endostatin. Li et al^21^ used gold nanoparticles (AuNPs) as a drug delivery system, which enhanced the concentration of rh‐endostatin in tumors.

In this study, we screened 136 patients with stage III/IV NSCLC, after PSM (cut‐off = 0.01) the selected 88 patients were administered 2 different doses of rh‐endostatin combined with platinum‐based chemotherapy or platinum‐based chemotherapy only, and their efficacy and safety were compared. The results revealed that the median PFS in the rh‐endostatin group and in the chemotherapy group was 6 months and 4.5 months, respectively (*P* = 0.047), reaching the primary endpoint. The median OS in the rh‐endostatin group was significantly longer (20 months vs 10 months, *P* < 0.001), and the DCR was significantly increased (83.3% vs 64.7%, *P* = 0.046). Our study firstly revealed rh‐endostatin plus chemotherapy to be better efficient compared with chemotherapy only group in real‐world practice, and provided evidence for the combined treatment. Rh‐endostatin plus chemotherapy should be recommended as first line standard treatment strategy for non‐driver gene mutation NSCLC patients. In our study, the platinum‐based chemotherapy regimen for each patient was different, including pemetrexed, gemcitabine, and paclitaxel combined with cisplatin or carboplatin. However, as shown in clinical trials of ECOG 1594 and JMDB, the efficacy and safety of all the 4 regimens were similar.^22^, ^23^ So, we think out data prove evidence to show rh‐endostatin combined chemotherapy improved the efficacy.

In the ECOG 4599 study,^24^ the median PFS in patients receiving paclitaxel/carboplatin combined with bevacizumab (15 mg/kg) was prolonged by nearly 2 months compared with those receiving chemotherapy only (6.2 months vs 4.5 months), the OS for the first time exceeded 1 year (12.3 months vs 10.3 months), and the ORR was significantly increased (35% vs 15%). While, in BEYOND study,^25^ the median OS in the experimental group was 24.3 months, which was 6.6 months longer than that of the control group (24.3 months vs 17.7 months, *P* = 0.015), the median PFS was prolonged by 2.7 months (9.2 months vs 6.5 months, *P* < 0.001)，and the ORR was also significantly increased (54% vs 26%). In our study, the median OS was 22 months and 14 months in the rh‐endostatin 14d group and the rh‐endostatin 7d group, respectively, which was both significantly, improved compared with results of the ECOG 4599 study (12.3 months). The median PFS in the rh‐endostatin 14d group (6 months) and the rh‐endostatin 7d group (6.5 months) were similar with the ECOG 4599 study (6.2 months), but less than BEYOND. In addition, the ORR in the rh‐endostatin 14d group of this study was higher than that in the ECOG 4599 study (44.4% vs 35.0%) but lower than BEYOND. The ORR in the rh‐endostatin 7d group (22.2%) was not as high as that in the ECOG 4599 study or BEYOND study (54.0%). This discrepancy may be due to the enrolled patients in the BEYOND study^25^ had higher proportion of women and non‐smokers who tended to which carried driver gene mutations, while in our study, all patients were negative for driver gene mutations. In safety analysis, severe adverse reactions in the rh‐endostatin group included leukopenia in 14 cases (25.9%), granulocytopenia in 9 cases (16.7%), anemia in 3 cases (5.6%), thrombocytopenia in 3 cases (5.6%), and nausea in 3 cases (5.6%). Additionally, their incidence (leukopenia: 25.9%; neutropenia: 18.5%) was slightly higher than those in the chemotherapy group. The incidence of severe vomiting was 3.7%, lower than that in the chemotherapy group (5.9%). Grade 4 adverse reactions occurred in only 1 patient of rh‐endostatin 7d group who developed thrombocytopenia after treatment, and platelet count gradually returned to normal after treatment of megakaryocyte (recombinant interleukin 11). Other adverse events associated with rh‐endostatin included 1 case of hemoptysis (1.9%) and 3 cases of cardiac toxicity (5.6%), no hypertension and proteinuria events were observed in our study. This was significantly lower compared with ECOG4599 and BEYOND.^24^ Our results partially showed that rh‐endostatin was safer. Our results partially showed that rh‐endostatin was safer compared with bevacizumab for side effect management.

We also compared the efficacy, survival time, and safety of the 2 different administration regimens of rh‐endostatin. The median PFS in the rh‐endostatin 14d group and the rh‐endostatin 7d group was similar (6 months vs 6.5 months) but statistically significant (*P* = 0.044). Although the OS was significantly longer in the rh‐endostatin 14d group than that in the rh‐endostatin 7d group, the difference was not of statistical significance (*P* = 0.111). In addition, although the ORR and DCR in the rh‐endostatin 14d group showed more advantage, there was no significant difference compared with the rh‐endostatin 7d group (ORR: *P* = 0.074, DCR: *P* = 0.234). Regarding the safety, the incidence of severe myelosuppression was similar in the rh‐endostatin 14d group and the rh‐endostatin 7d group, which was 55.6% and 51.9%, respectively. Likewise, the incidence of severe nausea and vomiting was also roughly equivalent in rh‐endostatin 14d group (7.4% and 0%) and rh‐endostatin 7d group (both 3.7%). However, among the grade 1‐2 adverse events, nausea, and vomiting events were more common in the rh‐endostatin 14d group. In the rh‐endostatin 14d group, mild nausea occurred in more than half (55.6%) of the patients, mild vomiting occurred in 6 patients (22.2%), while in the rh‐endostatin 7d group, mild nausea occurred in only 3 cases (11.1%) and no grade 1 or 2 vomiting occurred. Generally, there was no significant difference between 7‐day administration and 14‐day administration of rh‐endostatin; 7‐day and 14‐day for both efficacy and safety. Treatment of rh‐endostatin 7d will also be a reliable administration method for patients, and with less hospital stay time. Large sample size random trials should be conducted for further evaluation.

Certainly, there are limitations in this study, including the small sample size, and difficulties of some patients in the follow‐up period that led to truncated data. In conclusion, rh‐endostatin plus platinum‐based chemotherapy significantly improved the PFS, OS, and DCR in advanced non‐driver gene mutation NSCLC patients with well tolerance. The 7‐day administration regimen was not inferior to the 14‐day administration regimen of rh‐endostatin in efficacy and safety. The 7‐day administration regimen of rh‐endostatin needs further studies before its adoption in clinical work.

## CONFLICT OF INTEREST

None declared.
